# Supplementary material for: Overexpression of Barley Transcription Factor HvERF2.11 in Arabidopsis Enhances Plant Waterlogging Tolerance
Source: Int J Mol Sci. 2020 Mar 13;21(6):1982. doi: 10.3390/ijms21061982 (PMC7139581; doi:10.3390/ijms21061982)
Supplement: Supplementary file 1 [file ijms-21-01982-s001.zip › supplementals/Supplemental Data 1.docx]

**Supplemental Data 1:** **Protein sequences for phylogenetic and cluster analysis of ERF TFs with conserved N-terminal motif (MCGGAI/L) in Arabidopsis, rice and barley**

| **At2g47520 HRE2** |
| --- |
| MCGGAIISDFIWSKSESEPSQLGSVSSRKKRKPVSVSEERDGKRERKNLYRGIRQRPWGKWAAEIRDPSKGVRVWLGTFKTADEAARAYDVAAIKIRGRKAKLNFPNTQVEEEADTKPGGNQNELISENQVESLSEDLMALEDYMRFYQIPVADDQSATDIGNLWSYQDSN |
| **At3g16770 AtEBP/RAP2.3** |
| MCGGAIISDYAPLVTKAKGRKLTAEELWSELDASAADDFWGFYSTSKLHPTNQVNVKEEAVKKEQATEPGKRRKRKNVYRGIRKRPWGKWAAEIRDPRKGVRVWLGTFNTAEEAAMAYDVAAKQIRGDKAKLNFPDLHHPPPPNYTPPPSSPRSTDQPPAKKVCVVSQSESELSQPSFPVECIGFGNGDEFQNLSYGFEPDYDLKQQISSLESFLELDGNTAEQPSQLDESVSEVDMWMLDDVIASYE |
| **At1g72360 HRE1** |
| MCGGAVISDYIAPEKIARSSGKSSWRSNGVFDCSIYDFDGNFDELESDEPFVFSSTHKHHASGSASDGKKKQSSRYKGIRRRPWGRWAAEIRDPIKGVRVWLGTFNTAEEAARAYDLEAKRIRGAKAKLNFPNESSGKRKAKAKTVQQVEENHEADLDVAVVSSAPSSSCLDFLWEENNPDTLLIDTQWLEDIIMGDANKKHEPNDSEEANNVDASLLSEELLAFENQTEYFSQMPFTEGNCDSSTSLSSLFDGGNDMGLWS |
| **At1g53910 RAP2.12** |
| MCGGAIISDFIPPPRSRRVTSEFIWPDLKKNLKGSKKSSKNRSNFFDFDAEFEADFQGFKDDSSIDCDDDFDVGDVFADVKPFVFTSTPKPAVSAAAEGSVFGKKVTGLDGDAEKSANRKRKNQYRGIRQRPWGKWAAEIRDPREGARIWLGTFKTAEEAARAYDAAARRIRGSKAKVNFPEENMKANSQKRSVKANLQKPVAKPNPNPSPALVQNSNISFENMCFMEEKHQVSNNNNNQFGMTNSVDAGCNGYQYFSSDQGSNSFDCSEFGWSDQAPITPDISSAVINNNNSALFFEEANPAKKLKSMDFETPYNNTEWDASLDFLNEDAVTTQDNGANPMDLWSIDEIHSMIGGVF |
| **At3g14230.3 RAP2.2** |
| MCGGAIISDFIPPPRSLRVTNEFIWPDLKNKVKASKKRSNKRSDFFDLDDDFEADFQGFKDDSAFDCEDDDDVFVNVKPFVFTATTKPVASAFVSTGIYLVGSAYAKKTVESAEQAEKSSKRKRKNQYRGIRQRPWGKWAAEIRDPRKGSREWLGTFDTAEEAARAYDAAARRIRGTKAKVNFPEEKNPSVVSQKRPSAKTNNLQKSVAKPNKSVTLVQQPTHLSQQYCNNSFDNSFGDMSFMEEKPQMYNNQFGLTNSFDAGGNNGYQYFSSDQGSNSFDCSEFGWSDHGPKTPEISSMLVNNNEASFVEETNAAKKLKPNSDESDDLMAYLDNALWDTPLEVEAMLGADAGAVTQEEENPVELWSLDEINFMLEGDF |
| **Os09g0287000** |
| MCGGALIPNDYGDKPPPPPSESSEWDATTKMKKKKKRGGGGDDDWEAAFREFIAGDDDDDDGGVSMFPSGAGTMETTTEVAPAAAVVERPRRRRRVRRSYPYRGVRQRPWGRWASEIRDPVKGARVWLGTFDTAVEAARAYDAEARRIHGHKARTNFPPDEPPLPAPSQAPFCFLLDDDDDDDGVARGNSPASSSAPDRASACTTSSTVASGERGDELILLECCSDDVMDSLLAGFDVSSEPRSVLGMVN |
| **Os03g0182800** |
| MCGGAILADFTPARVPRRLTAAELLPVTPTPPAAERRTTRKRKSDVDFEAEFELFEDDDDDDEFELSDDGDESLAVSCVSSPKSKAVPSFSFSSDVSSSSRPRRRVAAAAAGRRKASKKSKYRGVRRRPSGRFAAEIRDPKKGRRVWLGTYGSAEEAAMAYDREARRIRGKGARLNFPRDGDGSPRRSNDRPCWTIDLNLPAAAVSGDDDDAMAVDAADADAGSAGRAAAYADQEALSAAKCKIKQCPRDEQMASATPELMEEDASSSRNMVPLSMALQLQYAAMIAECDREMEEIAAVERDLERRRRQVFERRGHLVRQASLLLD |
| **Os03g0341000** |
| MCGGAIPLISSRGPGGKRSLSAADELWPPPPQHASDDPAEQAAADEEEQEQQPAARRQRRGERRTLYRGIRRRPWGKWAAEIRDPAKGARVWLGTFATAEAAARAYDRAARRIRGTKAKVNFPNEDNAFAAAPPPYHLAAYYGDASSTSYLYPMAMTPAAAGLREQQLMTTTAVEYSVNDAVDVASVYFQPPPPAVAYEFSAVGGGAVVVPVSAVAPAMTYGQSQEVAAPLMWNFDDITAMPM |
| **Os07g0674800** |
| MCGGAIISDFIPQREAHRAATGSKRALCASDFWPSASQEAADFDHLTAPCTFTPDQAAEEPTKKRERKTLYRGIRRRPWGKWAAEIRDPAKGARVWLGTFATAEAAARAYDRAARRIRGAKAKVNFPNEDPPLDDPAADGHSHGGAAIPCREFMDYDAVMAGFFHQPYVVADGVPAVPAEEAPTVAYVHHHLPPQPQQDAGLELWSFDNIHTAVPM |
| **Os01g0313300** |
| MCGGAIIYDYIPARRRLCASDFWPDADDSDPHTPAPEKPPRAKRERKNQYRGIRQRPWGKWAAEIRDPVKGVRVWLGTYPTAEAAARAYDRAARRIRGAKAKVNFPNDFGAAPAPAAAAAKAVPRVAPTPAVLPPPKMEAVSEGAGACSSDEVKELSEELLAYENYMSFLGIPYMEGGAASAAGAEEAAAPAGLWTFEDYELPSLAL |
| **Os05g0361700** |
| MCGGAIIADFVPPAGARRAAASDISDNAVLSAAGAGDESFAAAKAPAPGRKTAYRGIRRRPWGRWAAEIRDPRKGARVWLGTYATAEEAARAYDVAARDIRGAKAKLNFPPTIGAAAAPPPPKKRRKAAAAANHHHHHHQQESSGSSSASSLPPTPPPAAEHQLRECMSGLEAFLGLEEEEDDGGAGEPWDAVDMMLE |
| **Os03g0183000** |
| MCGGAILAEFIPAPSRAAAATKRVTASHLWPAGSKNAARGKSKSKRQQRSFADVDDFEAAFEQFDDDSDFDDAEEEDEGHFVFASKSRVVAGHDGRAAARAASKKKRGRHFRGIRQRPWGKWAAEIRDPHKGTRVWLGTFNTPEEAARAYDVEARRLRGSKAKVNFPATPAAARPRRGNTRATAVPPPATAPAAAPPRGLKREFSPPAETALPFFTNGFVDLTTAAAPPPAMMMTSSFTDSVATSESGGSPAKKARSDDVDSSEGSVGGGSDTLGFTDELEFDPFMLFQLPYSDGYESIDSLFAAGDANSANTDMNAGVNLWSFDDFPIDGALF |
| **Os07g0617000** |
| MCGGSILGDLHLPVRRTVNAGDLWGDAGKGRDGGDGLKKRKGSSWDFDVDCDDDDDDDFEADFEEFEDDYGDDDDVGFGDDDQESDMNGLKLAGFSTTKLGLGGSRKRKTRYRGIRQRPWGKWAAEIRDPRKGVRVWLGTFGTAEEAAMAYDVEARRIRGKKAKVNFPDAAAAAPKRPRRSSAKHSPQQQKARSSSSSPASLNASDAVSKSNNNRVSSAGSSTDATAAAIAIDDGVKLELLSETDPSPPMAAAAAAWLDAFELNDLDGSRCKDNAFDHQIHKVEAAVADEFAFYDDPSYMQLGYQLDQGNSYENIDALFGGEAVNIGGLWSFDDMPMEFRAY |
| **Os09g0434500** |
| MCGGAIISGFIPPSAAAAAAAAVAKKQQGRRVTADVLWPGMLRKGKAAAAEEDFEADFREFERGMSDDEAEGGGGEEEEDDDDVVVVVPPPAAARFVVRAAAKAAPPTADGMLTTKLVQHDGPTARSAKHKRKNQYRGIRQRPWGKWAAEIRDPSKGVRVWLGTYNTAEEAARAYDAEARKIRGKKAKVNFPDEPAVAQKLSLKQNAAKQEKLAPPLKTCGDDAFFQLNSSDNDLFAMLAKVPAKPAEPVDLMPPVKPLASTETFEMNMLSDTSSNSFGSSDFGWEDDTLTPDYTSVFVPNAAMPAYGEPAYLTGGAPKRMRNNYGIAVPQGNGMPNLAQNMPTFDPEMKYLPLPYVESSSDESMDNLLQNDATQDGASNEGIWSLDELLMAAGAY |
| **Os06g0194000** |
| MCGGAILSDLIPPPRRVTAGDLWLEKTKKQQQQKKKNKGARRLPLRQEEEDDFEADFEEFEVDSGEWEVESDADEAKPLAAPRSGFAKGGLKNTTVAGADGPAARSAKRKRKNQFRGIRQRPWGKWAAEIRDPRKGVRVWLGTFNSPEEAARAYDAEARRIRGKKAKVNFPDGAPVASQRSHAEPSSMNMPAFSIEEKPAVMSAGNKTMYNTNAYAYPAVEYTLQEPFVQIQNVSFVPAMNAIEDTFVNLSSDQGSNSFGCSDFSQENDIKTPDITSMLAPTMTGVDDSAFLQNNASDAMVPPVMGNASIDLADLEPYMKFLIDGGSDESIDTLLSSDGSQDVASSMDLWSFDDMPVSAEFY |
| **Os02g0782700** |
| MCGGAIIHHLKGHPEGSRRATEGLLWPEKKKPRWGGGGRRHFGGFVEEDDEDFEADFEEFEVDSGDSDLELGEEDDDDVVEIKPAAFKRALSRDNLSTITTAGFDGPAAKSAKRKRKNQFRGIRQRPWGKWAAEIRDPRKGVRVWLGTFNSAEEAARAYDAEARRIRGKKAKVNFPEAPTTAQKRRAGSTTAKAPKSSVEQKPTVKPAFNNLANANAFVYPSANFTSNKPFVQPDNMPFVPAMNSAAPIEDPIINSDQGSNSFGCSDFGWENDTKTPDITSIAPISTIAEVDESAFIKSSTNPMVPPVMENSAVDLPDLEPYMRFLLDDGAGDSIDSLLNLDGSQDVVSNMDLWSFDDMPVSDFY |
| **Os03g0183300** |
| MCGGAILAELIPSAPAARRVTAGHVWPGDANKAKKKGARADDFEAAFRDFDNDSDDEEMMVEEAEEEEATSEHKPFVFRAKKAAAAASSRRRKPAQYRGVRRRPWGKWAAEIRDPVKGIRVWLGTFTNAEAAALAYDDAARAIRGDRAKLNFPSATTPDTRKRGRATAAAAPAVKATPVINLVEEEDEEEVAAAMASIKYEPETSESSESNALPDFSWQGMSASDEFAVAAAALSLDSDDDLAKKRPRTEPEDTTDSGSGDDTDALFDALLFADQYNHFNGGAYESLDSLFSADAVQTTAAAAAADQGMGLWSFDDGCCLVDVEASLSF |
| **Os10g0390800** |
| MCGGAILADLIPSPRSGGHTKKNKRRRISDDEDFEAAFEEFDAGDDDSDSDSESEEVDEYDVVVDDDDSEDGVVVLPPPPPPPPVIPHERHGARRFRGVRKRPWGKWAAEIRDPVRGVRVWLGTFPTAESAARAYDAAARRLRGAKAKPNFPSAPPPSAAAHRRKKRRAHAATRSPSSPPATSEVTAASASASSDVPAPAFASFVGEPGHGGAKSMPTTSHTSQPAPPATVASENVDDPEVFDPYDVHGGLASYFAGGAYESLESLFAHGGDSAAVDQAASDHWPAALWSFADDGSFCF |
| **Os03g0183200** |
| MCGGAILANIIPATPRPRKCRPTTATATPKATTPNVVVVVNLVDKEAEVSESSGASSSALPDFSWQGMSASSDDDAAAQQALLDAAGGAKKRPRSEPHVTSDDEVLPASFDSDNNTAAAGLLPLDDPFLFGDQFGDLNGGAFASLMDGLFAAGEANVAGESVGLWSFGDDFLNASYY |
| **OsSUB1A-1** |
| MCGGEVIPADMPAAPFTPRHGDGETWVDRKRRNKKKRKRGADEEWEAAFQEFMAADDDDDGGGLVLSSKSLVLRSPGENDAGRGAAATMSMPLDPVTEEAEPAVAEKPRRRRPRRSYEYHGIRQRPWGRWSSEIRDPVKGVRLWLGTFDTAVEAALAYDAEARRIHGWKARTNFPPADLSSPPPPSQPLCFLLNDNGLITIGEAPTDDAASTSTSTTEASGDARIQLECCSDDVMDSLLAGYDVASGDDIWTWTSGASSTSVNQEIKTPSIHQNISYAGEA |
| **OsSUB1B-1** |
| MCGGALIPNDYGDKPPPPPSESSEWDATTKMKKKKKRGGGGDDDWEAAFREFIAGDVDDDDDGVSMFPSGAGTMETTTEVAVVERPRRRRRVRRSYPYRGVRQRPWGRWASEIRDPVKGARVWLGTFDTAAEAARAYDAEARRIHGHKARTNFPPDEPPRPAPSQAPFCFLLDDDDDGVARGNSPASSSAPDSTSACTTSSTVASGERGDELILLECCSDDVMDSLLAGFDVSSESRSILGMVN |
| **OsSUB1C-1** |
| MRRRVSSSPSSSSSSSPARHHKARRSRRKLVADEDWEAAFREFLSRDDDDDDDDDDGHHVVVAPLIRSSNKCVHGHEVVASTVGGGASGGRRRADDDDGERRRRRRRERRSYPYRGIRQRPWGRWASEIRDPVKGIRVWLGTFDTAEGAARAYDDEVRRIYGGNAKTNFPPSPPPPEQPAAPVAAERSPSTTTTTTPSAEDSGDSRILIECCSDDLMDSLLAAFDMTTGDMRFWS |
| **OsSNORKEL1** |
| MCGGCLIPDELVGKPARRTRAAAAGGDSGDGWKHGRRLCPAAAPCNCKPRRRAGAADDDDVGRRRRTTRTRAASEVRFHGIHMRSYGRWSAEIRDSSYRGHRLWIGTYATAEAAARAYDAEARRIHGAKANTNFPPPPNDVDSGAPPPPPWDLEAHMRFLGEVELDDGGAEPPPPPSYGIPELLHMEPELASATQSVHGDDEPWGLDKYMRFLSEVELDDGGAPLPPPPSQHGGVAAAGSPQYGCRYDYLLLMMCN |
| **OsSNORKEL2** |
| MCGENDNNGAAAGSSRRLPAVGAMRGPCIEEKLKTVVVVLSDDDDDYEEEFRRYCENTTLPDKGDKGGRRRPAASKKQHRHRFHGIHRRKSGRWSAEIRDNMIKGSRSWVGTFYTAEEAAWAYDAVARRLYGPNARTNFPLPPPPPPPVAPLLPAPAVANKKMNSKSKKPAPKMVVAPAGGETAAAAGEMAPVLLGNALEASNGWEFEPYSCMGLVVCSAVYNYADEPEPADDELQLLHLMHGGAMADFAADGCLWSF |
| **HvERF2.3 AK251681.1** |
| MCGGAILKDLKVPAVTRKVTEAALWPEKKKPRQADGGARRLGLVDGEEDFEADFEEFEADSGDSDLELGRGRAAEKDDDEVVEIKPYAAVKRPLSQDDFSIITTAGCDGPAQRSAKRKRKNQFRGIRQRPWGKWAAEIRDPSKGVRVWLGTFNSAEEAARAYDVEARRIRGKKAKVNFPEEPTVPQKRRVRPAPLKAPKLSASQEPTVIPAVNNLANPNAFVYPSADFASNQPPVQPDNVPFVPAMKFAAPVEAPVMNMYSDQGSNSFGCSDLGWDYETKTPDISSVAPISTIAEGAESALIQSNTYNSVVPPVMENNAVDFEPWTRFLMDDGVDEPIDSLLNFDVPQDVVSNMDLWSFGDMPICGEFF |
| **HvERF2.4 AK357064** |
| MCGGAILSDIIPPPRRAAGGRLWQADRKKRRAGPRRVPEEEPEEEAEEGDEDFEADFEGFVDEESDGEVKPFPARRSGFSGDGLKATAAGEYDCASGSAKRKRKNQFRGIRRRPWGKWAAEIRDPRKGVRVWLGTYNSAEEAARAYDVEARRIRGKKAKVNFPEEAPMASQQRCPEPTAVKVPEFNTEQKPVLNTMGNADVYSCAAVDYTLNQQFVQPQNMSFVPTMNAVEAPFMNFSSDQGSNSFSCSDFSWENDIKTPDITSVLASIPTSTEVNESAFVQNNGSNSTAPPVMGNANVDLADLEPYMKFLMDDRSDESIDSILSCDVPQDVVGNMDLWTFDDMPLSAGFY |
| **HvERF2.6 MLOC_58276.1** |
| MCGPLTHKNVDVPPVTRKVMAATFWPEKKKPRQTDGRGRHFLGLGGLRGLGLDEKEEDFEADFKEFEVNFGDSDLELGHNGVIGKDDHEDVVEIKPFVVIKGFLSQDDLSTMSTAGPSERPAKRKRKNQFRGTRQRPWGKWAAEIRDPSKGVRLWLGTFRSAEEAARAYDVAARRIHGKKAKVNFPEEPAVEEPTVIPALNNLANPNAFVYPSVDFASNQPLVQIANMPFVPAMNFVAPVQAPAMNMYSDQGSNTFGCSDLGRQYTIKTPDMSSIIAPVSTIAEGAESARVQSNTYNPVVIAEAAESALVQSNTYNSVVPSIMESDSVDFDAWTRFLMDDDVDEPIDSLVNFDVLQDVLGNMDLWNFDDMPICG |
| **HvERF2.7 MLOC_59004.2** |
| MCGGTNLEDVEVPVVTRKLTEATPWIEKKKPRQSDGKGRHFTRLRGLRGLGLDDDEEDFEADFKEFQANSWDSDQELERDGVGEKDDDDEVVEIKPFSIVKRSLSQDDLSTMSTFGFDGPSERPAKRKRKNQFRGTRQRPWGKWAAEIRDPSKGVRLWLGTFRSAEEAARAYDVAARRIHGKKAKVNFPEEPAVQEPTVIPALNNLANPNDFIYPSVDFASNQPLVQTANMPFVPEMNFVAPVQAPVMNMYSDQGSNTFGCFDLGRQYTIKTPVSTIAEGAESALVQSNTYNPVVIAEGAESAFVQSNTYNSVAPPVMKSDGVDFDAWMRFFLDDDVDEPIDSLVNFDVLQDVLGDMDLWNFDDMPICG |
| **HvERF2.11 AK372481** |
| MCGGAILAGFIPPSAAAAAAKAAATAKKKQQQRSVTADSLWTGLRKKADEEDFEADFRDFERDSSEEEDDEVEEVPPPPAPATAGFAFAAAAEVALRAPARRDAAVQHDGPAAKQVKRVRKNQYRGIRQRPWGKWAAEIRDPSKGVRVWLGTYDTAEEAARAYDAEARKIRGKKAKVNFPEDAPTVQKSTLKPTAAKSAKLAPPPKACEDQPFNHLSRGDNDLFAMFAFSDKKVPAKPTDSVDSLLPVKHLAPTEAFGMNMLSDQSSNSFGSTDFGWDDEAMTPDYTSVFVPSAAAMPAYGEPAYLQGGAPKRMRNNFGVAVLPQGNGAQDIPAFDNEVKYSLPYVESSSDGSMDNLLLNGAMQDGASSGDLWSLDELFMAAGGY |
| **HvERF2.13 AK367417** |
| MCGGAIIYDYIPAAAHRRRASTADFWHDANDHSDAYSAAPDKAPRAKRGRKNQYRGIRQRPWGKWAAEIRDPVKGVRVWLGTYPTAEAAARAYDRAARRIRGAKAKVNFPNEILAGAPAHQASCTMAAAPPPAALPSPKKEEGVEPAAPCSCEEVKALSEELMAYESYMSFLGVPYMEGGAAAAVGAAAEEAPAELWSFEDSYYYPGPLGL |
| **HvERF2.14 MLOC_64636.1** |
| MCGGAVIADFVPAAARRPDGSSTDVPGSSLTGEEVTEKAPAPARKTAYRGIRRRPWGRWAAEIRDPRKGARVWLGTYATAEEAARAYDVAARDIRGVKAKLNFPPAVGAPQAAVAGAPKKRPRVAAEESSASWSPLPGTATGVGSTDSLRESMSGLEAFLGLKDAAGDDDVQPWEAVDIIF |
| **HvERF2.17 AK367525** |
| MCGGAILAELISPSAGRAPKQAQVAAAGPVSANKDGKSKGHKHNKYGSVADVDDDLQDFDDDLDLRQAEEDADDHVVFACKPAFSPGPAYDGGRAAQAASRKKKRVLHGIRQRPWGKWAAEIRDPHKGTRVWLGTFDTADDAARAYDVAARRLRGSKAKVNFPDAARAGARPRRASRRTAQKPQRPPAWTTAYSATAAAHSQPEQDAFVDLTTAVTALPPIMESSFADSGSTKPMFHEDSSAGSGGGAMPGFTDELGFDPFMLFQLPCSDTYESIDSLFAGDAALQDARGVDSGIDGVSLWSFDEFPMDSAIF |
| **HvERF2.18 AK364086** |
| MCGGAILAELIPGAPARRVTPGHIWPGKGAKQAKPAAAADDFEAAFREFNEEEDEEEEVVVERQEEVAESKPFVFAASPKKQLRQEEAAPSRSRKPSQYRGVRRRPWGKWAAEIRDPVKGVRVWLGTFPSAEAAALAYDDAARAIRGPRAKXNFPSSAVAAAPGARKRGRAGVALAANAKPVIHIIDEEEEHAALAAPASSFVKHEAEASEGSESSGALPDFSWQGMSAFDEAPAYPAPEPETELPAKRARTEPEDTDEGMSAHPASDSDSDALFDALLFADQFAYFNGGAYESLDSLXSADAVQSSTATAAGEAAMGLWSFDDDCLVDECSLSF |
| **HvERF4.7 MLOC_68290.2** |
| MCCGAEVADPGEHRRRLAAGDDDGGDGREEHALSPPTANTTTMWRRRQAQEMSAMVSALAQVVAGGRGGGNEGGMAMASSAKRPAEHEAFTEEAWWSTSYGDGDGDGGARSASSFLAATPAASYSAYGAAAGDEELPSPSSAESGGGGGTPRKRYRGVRQRPWGKWAAEIRDPHKAARVWLGTFDDAEAAARAYDAAALGFRGSRAKLNFPESATIPPQQQPPPAPASAMPPPQRPEALLESQAYSHSHYARFLVPSTGEPSLLPAGSTPRTPLIYSFGAGASYPLEPESRGEGTAGTECPAGPDGDLSSGVQP |
| **HvERF4.8 AK365834** |
| MCFEFELADQRGPHGSGRAAKGGDGAAPEGMTTMASPADLMSGYYQAQEMSTMVSALSRVVAEDDPWAAASGSGSGAEGWGWEEQAMHAGAGGGYVHELGGFPSSEFAGSDQSSDTQGQSAATMEEHRSRSPAASNAAAEAEAPRRRYRGVRQRPWGKWAAEIRDPHKAARVWLGTFETAEAAARAYDDAALRFRGSRAKLNFPEDARLHPPTVAPAAAPIAAGSASPAVYTAGASQASDYLRYQMLLQSRGAAANQGNLLSFYGGADGGGMSSSYGGGGDGGAMSGFLGSYYSFPSSAVSVATVPSSSSASSAPGHYYSSSHDSQQQQQQQGDAAAEWSWESALAPYPGTAAAASWSGSSQQQYHPPHTQ |
| **HvDREB1.8 AK374663** |
| MCQIKKEMSGESGSPCSGENYYYSPSTSPEHQQAKQQAAWTSAPAKRPAGRTKFRETRHPVYRGVRRRGNAGRWVCEVRVPGRRGSRLWLGTFDTAEAAARANDAAMLALAAGGAGCLNFADSAELLAVPAASSYRSLDEVRHAVVEAVEDLLRREAHAEDDALSVSCTSSSAPSSLTDDESSSSPAAEGSPFELDVLSDM  GWDLYYASLAQGMLMAPPASLAAALGDYGEAHLADVPLWSYQS |
| **HvAP2-16 MLOC_8430.1** |
| MCGKVPLANSKISAVTRNVMEVTVLPEKNKPVWGSRGDRCFTGHGRRKGLRMDNDEEDFEAGFGDSNMELVRGGVAQKDDGNESLSQDDLSIMPTADFDGPSEMPKRRKRKNQFQGIRQRPLGKWAAEITHPTKDLHVWPDTFNSAEEAARGYDAEARRIHGKKAKVNFTEKATRSTDFASNQPLVPAMNSTAPVEAPVMDMYSDQGSNSFGSSDLGWEYDAKTPDISSIAPTSTIAEGEEFALVKNNTYNSMVPHVMENNGVNLQPWMRYLLDDSVDKMIDSLLNFDVPQDTIVNMDLWSFDDMPTGGEFF |
