# Supplementary material for: Overexpression of Barley Transcription Factor HvERF2.11 in Arabidopsis Enhances Plant Waterlogging Tolerance
Source: Int J Mol Sci. 2020 Mar 13;21(6):1982. doi: 10.3390/ijms21061982 (PMC7139581; doi:10.3390/ijms21061982)
Supplement: Supplementary file 1 [file ijms-21-01982-s001.zip › supplementals/Supplemental Data 2.docx]

**Supplemental Data 2 Protein sequences for phylogenetic and cluster analysis from *Hordeum vulgare* and other plant species**

**Hordeum vulgare (BAK03679.1)**

MCGGAILAGFIPPSAAAAAAKAAATAKKKQQQRSVTADSLWTGLRKKADEEDFEADFRDFERDSSEEEDDEVEEVPPPPAPATAGFAFAAAAEVALRAPARRDAAVQHDGPAAKQVKRVRKNQYRGIRQRPWGKWAAEIRDPSKGVRVWLGTYDTAEEAARAYDAEARKIRGKKAKVNFPEDAPTVQKSTLKPTAAKSAKLAPPPKACEDQPFNHLSRGDNDLFAMFAFSDKKVPAKPTDSVDSLLPVKHLAPTEAFGMNMLSDQSSNSFGSTDFGWDDEAMTPDYTSVFVPSAAAMPAYGEPAYLQGGAPKRMRNNFGVAVLPQGNGAQDIPAFDNEVKYSLPYVESSSDGSMDNLLLNGAMQDGASSGDLWSLDELFMAAGGY

**Aegilops tauschii (**[**XP 020194933.1**](https://www.ncbi.nlm.nih.gov/protein/XP_020194933.1?report=genbank&log$=prottop&blast_rank=1&RID=5YY9E20D016)**)**

MCGGAILAGFIPPSAAAAAAKAAAAKKKQQQRSVTADSLWPGLRKKAAEEEDFEADFRDFERDSSDDDAVVEEVPPPPASAGFAFAAAAEVAPPAPARLDAVQHDGPAAKQVKRVRKNQYRGIRQRPWGKWAAEIRDPSKGVRVWLGTYDTAEEAARAYDAEARKIRGKKAKVNFPEDAPTVQKSTLKPTAAKSAKLAPPPKACEDEPFNHLSRGDNDLFAMFAFNDKKVPAKPAESVDSLLPVKPLVPTETFGMNMLSDQSSNSFGSTDFGWDDEVMTPDYTSVFVPNAAAMPAYGEPAYLQGGAPKRMRNNFGVAVLPQGNVAQDIPAFDHEMKYSLPYVESSSDGSMDSLLLNGAMQDGASSGDLWSLDELFMAAGGY

**Triticum aestivum (AFP49824.1)**

MCGGAILAGFIPPSAAAAAAKAAAAKKQQQQQQQQQRSVTADSLWPGLRKKPAEEEDFEADFRDFERDSNDDDDAVEEVPPPPATAGFAFAAAAEVALPAPTRLDAIQHDGPAAKSVKRVRKNQYRGIRQRPWGKWAAEIRDPSKGVRVWLGTYDTAEEAARAYDAEARKIRGKKAKVNFPEEAPTVQKSTLKPTAVKSAKLAPPPKTCEDEPFNHLSRGDNDLFAMFAFNDKKVSAKPAESVDSLLPVKPLVPTETFGMNMLSDQSSNSFGSTDFGWDDEAMTPDYTSVFVPNAAAMPAYGEPAYLQGGAPKRMRNNFGVAVLPQGNGAQDIPAFDHEMKYSLPYVESSSDGSMDSLLLNGAMQDGASSGDLWSLDELFMAAGGY

**Brachypodium distachyon (XP 00357816.1)**

MCGGAILSGFIPPSAAAAAAAAKKKKQQQQRVMADALWPGLQRKAPQAEVEDFEADFREFERDSSEEDAGGDDDDVVEVVPPPPAKAGFAFAAVAEGALPPTVDAVTIPKSVEHDGSGTGPVKRNRKNQYRGIRQRPWGKWAAEIRDPSKGVRVWLGTYNTAEEAARAYDAEARKIRGKKAKVNFPDEEPTSWKSTVKTIAQKSIQKATATNSAKLTTPPNTCDDESFSHLSNADNDLFAMFAFDDKKVPAKPDESGDFLTQAKPLVPTETFGMNMLSDQSSNSFGSTDFGWDDEAMTPDYTSVFVPDAPYSKVAYLEGGAPKRMRINFGVDLSPQGNDAPNLAQDISVFDPEMKYLPLPYVGSSSDRSMDNLVVNDVMQDGASNVDVWGLDELLMAAGAY

**Oryza sativa (AAV98700.1)**

MCGGAIISGFIPPSAAAAAAAAAAAAKKQQGRRVTADVLWPGMLRKGKAGAAEEDFEADFREFERGMSDDEAEGGGGEEEEEEEDDVVVEVPPPATARFVVRAAAKAAPPTADGMLTTKLVQHDGPTARSAKRKRKNQYRGIRQRPWGKWAAEIRDPSKGVRVWLGTYNTAEEAARAYDAEARKIRGKKAKVNFPDEPAVAQKLSLKQNAAKQEKLAPPLKSCGDDAFFQLNSSDNDLFAMLAKVPAKPAEPVDLMPPVKPLASTETFEMNMLSDTSSNSFGSSDFGWEDDTLTPDYTSVFVPNAAMPAYGEPAYLTGGAPKRMRNNYGIAVPQGNGMPNLAQNMPTFDPEMKYLPLPYVESSSDESMDNLLQNDATQDGASNEGIWSLDELLMAAGAY

**Setaria italic (XP 004956913.1)**

MCGGAILSGFIRPSGAAAAAAKKQQQQQPRRVTADLLWPGLGSRKGALGEQDFEADFREFVRGLGEDGGDADAAGDDDDEVQEVPPPEPAMFTFAAAAKAAPTAADDVMTPKPVQHDEPTATSAKRNRKNQYRGIRQRPWGKWAAEIRDPSKGVRVWLGTYNTAEEAARAYDAEARKIRGKKAKVNFPDEEQDAQKSILKPTTANPTKLAPPTETCADEAFNNLNNGDNDLFAMFAFSDSKAPVKPAEIASFLPAVRPHVPTKRSASNMLSDQSSNSYGSSDFGWDDETMTSDYTSVFAPNNAVPAYADPACLQGEVSKRMRNTYGLAVPQGNGAPNLAQDMSGFNPEMNYLPLSYVESSPDTSMDSLLQNDAPQDGASTGDLWSLDEMLMAAGAF

**Sorghum bicolor (XP 021309361.1)**

MCGGAILSGFIPPSGVAAAAAAAKKKQQQGRRVTADLLWPGPGKKGALPQEEEDDFEADFREFERGLSEDDDVDGAGEGGDDEVQELAPPEPVRFAFAAAAKAPRPAVDGVMTPKDVQGDRPTANSVKRNRKNQYRGIRQRPWGKWAAEIRDPSKGVRVWLGTYNTAEEAARAYDAEARKIRGKKAKVNFPDEVPGAQKSTAKPTDSNATKLAPPPKACADEVFSNLNNDNNDLFAMFAFNDNKVPVKPAEVASFLPAVKPLVPSKRSATNMLSDQNSNSYGSSDFGWDDDTMTSGYTFAPNNAIPASYMQGGESKRMRNNYGVAMFQGNGAPSLAQVMPGFDPEMNYQPLPYVESSSSDASMDSLLPQDGASNGDIWSLDELLMAAGAY

**Zea mays (NP 001149434.1)**

MCGGAILSGFIPPSGVPAAAAAAKKKQQRRATADLLWPGPGKKGAPREEDFEADFREFERGLGEDDDVDGAGDEVQELPLPEPARLAFAAAVGAPRTAVDGVMTPKDGEGDMPTTATNSAANKRRRKNQYRGIRRRPWGKWAAEIRDPSKGVRVWLGTYSTAEEAARAYDAEARRIRGKKAKVNFRDEAAAAAGAQKAPAATTPTACGNAARLGPPPPPPKFRADEVFGNMNGGTGSNDLFAIMFAFSDSSSKVVRVEPGEGAAGFLPADLLPGSKRSAANMLLLSDQSSDSYGSCDLGWEWDWDDDTMTSDYASVFAPAAPSNVVPAWYTQGGPVSKRTRSSYGYGAAMPGGFDPETNYQYQPLPYVVESSPSDGASTDDMDCLQMMRAGDVPQDGASSGGGGGDGGDIWSLDELLMAAGAY
