# Supplementary material for: Overexpression of Barley Transcription Factor HvERF2.11 in Arabidopsis Enhances Plant Waterlogging Tolerance
Source: Int J Mol Sci. 2020 Mar 13;21(6):1982. doi: 10.3390/ijms21061982 (PMC7139581; doi:10.3390/ijms21061982)
Supplement: Supplementary file 1 [file ijms-21-01982-s001.zip › supplementals/Table S1.docx]

**Table S1** qRT-PCR and cloning primers used in this study

| Primer name | Primer sequence | Description |
| --- | --- | --- |
| HvERF2.11-F | 5'-AAAGCTCGCCTCCTCCATAC-3' | Gene cloning |
| HvERF2.11-R | 5'-CAGGCATATGACCCAAGGTG-3' |  |
| Gate-25-F | 5'-GGGGACAAGTTTGTACAAAAAAGCA  GGCTTCATGCCGCCCGCAGCCATGGC-3' | Vector construction |
| Gate-25-R | 5'-GGGGGACCACTTTGTACAAGAAAGCT  GGGTCCTACTCGTTTGCGGCGGCGG-3' |  |
| HvERF2.11-F | 5'-TGGGAACCGTATGGTCATGTTT-3' | qRT-PCR |
| HvERF2.11-R | 5'-CAGGCATATGACCCAAGGTG-3' |  |
| Hvactin-F | 5'-GGTCCATCCTAGCCTCACTC-3' | qRT-PCR |
| Hvactin-R | 5'-GATAACAGCAGTGGAGCGCT-3' |  |
| AtSOD1-F | 5'-GTTGGTAGGGCTGTTGTTGTC-3' | qRT-PCR |
| AtSOD1-R | 5'-TGGACCTCCTTATTACATCAA-3' |  |
| AtCAT1-F | 5'-AGCGCTTTCGGAGCCTCGTG-3' | qRT-PCR |
| AtCAT1-R | 5'-GGCCTCACGTTAAGACGAGTTGC-3' |  |
| AtPOD1-F | 5'-GCACATACGATAGGAGTCACACA-3' | qRT-PCR |
| AtPOD1-R | 5'-GACAAGCAACACGCAAGAAC-3' |  |
| AtADH1-F | 5'-TTGCTCCACCGCAGAAACAC-3' | qRT-PCR |
| AtADH1-R | 5'-CCAACACTCTCAACAATCCCTCC-3' |  |
| AtPDC1-F | 5'-TGATGCTTCAGGCTATGCTTT-3' | qRT-PCR |
| AtPDC1-R | 5'-GTTGAAGATTGGACCTGCAAA-3' |  |
| AtACO1-F | 5'-CCAGTCAGAGATGGTCAAGG-3' | qRT-PCR |
| AtACO1-R | 5'-CATCCATCGTCTTGCTGAG-3' |  |
| Atactin-F | 5'-CCAACAGAGAGAAGATGA-3' | qRT-PCR |
| Atactin-R | 5'-ATGTCTCTTACAATTTCCCG-3' |  |
| AtACT8-F | 5'-TGCAGACCGTATGAGCAAAG-3' | qRT-PCR |
| AtACT8-R | 5'-CCGTCATGGAAACGATGTCT-3' |  |
